# High expression level of MMP9 is associated with poor prognosis in patients with clear cell renal carcinoma

**DOI:** 10.7717/peerj.5050

**Published:** 2018-07-04

**Authors:** Haitao Niu, Feng Li, Qingshui Wang, Zhoujie Ye, Qi Chen, Yao Lin

**Affiliations:** 1 Fujian Normal University, Fujian Key Laboratory of Innate Immune Biology, Biomedical Research Center of South China, College of Life Sciences, Fuzhou, China; 2 Provincial Clinical Medical College of Fujian Medical University, Department of Pathology, Fuzhou, China; 3 Fujian Normal University, Provincial University Key Laboratory of Cellular Stress Response and Metabolic Regulation, College of Life Sciences, Fuzhou, China

**Keywords:** MAPK, MMP9, ERK, ccRCC

## Abstract

Matrix metallopeptidase 9 (MMP9) was found to be associated with tumor aggressiveness. In this study, we focused on the correlation between MMP9 expression and clear cell renal carcinoma (ccRCC). Through the Gene Expression Omnibus (GEO) database, the Cancer Genome Atlas (TCGA) database and immunohistochemical (IHC) staining, we observed that compared with adjacent normal renal tissues, in ccRCC tissues the mRNA and protein levels of MMP9 were enhanced, and the mRNA levels of GTP-binding protein smg p21B(RAP1B), B rapidly accelerated fibrosarcoma (RAF), methyl ethyl ketone2 (MEK2), extracellular regulated protein kinases1 (ERK1), ERK2, v-ets avian erythroblastosis virus E26 oncogene homolog1 (ETS1) and ETS2 also increased. The Kaplan–Meier survival analysis suggested that high MMP9 expression was an unfavorable prognostic biomarker for ccRCC patients. Our results indicated that the increased expression level of MMP9 in ccRCC may be due to the activation of the Mitogen-activated protein kinases (MAPK)/ERK signaling pathway, and MMP9 may be an attractive target for ccRCC therapy.

## Introduction

Renal cell carcinoma is the most common malignant tumor derived from kidney tissue. At present, clear cell renal cell carcinoma (ccRCC) is the major histological subtype of renal cell carcinoma, accounting for 80–90% of cases (Forsea et al., 2012; Ljungberg et al., 2010). The formation and metastasis of ccRCC is a complex and continuous process with the participation of a number of key genes. Matrix metalloproteinases (MMPs) proteins participate in some physiological processes such as cell migration, angiogenesis, embryonic development, reproduction and so on (Vandooren, Pe & Opdenakker, 2013; Wang & Tsirka, 2005). MMPs-mediated degradation of extracellular matrix is a crucial event for invasion and metastasis of malignant cells. The expression of MMPs is elevated in some carcinomas and accelerates tumor progression. MMP9, a member of the MMPs, plays an important role in extracellular matrix remodeling and angiogenesis, which have been linked to the aggressiveness of gynecological tumors such as cervical cancer (Rauvala et al., 2006), endometrial Osteosarcoma . (Sun et al., 2018) and ovarian carcinoma (Brun et al., 2008; Ozalp et al., 2003; Schmalfeldt et al., 2001).

The expressions of MMP9 are regulated by different cytokines and growth factors. The Mitogen-activated protein kinases (MAPK) signaling pathway, one of the important pathways in eukaryote signaling network, plays a key role in gene expression regulation and cytoplasmic function. The MAPK/methyl ethyl ketone (MEK) pathway (also known as the rat sarcoma (RAS)/rapidly accelerated fibrosarcoma (RAF)/MEK/extracellular regulated protein kinases (ERK) pathway) is a chain of proteins in a cell that communicates a signal from a receptor on cell surface to the DNA in the nucleus (Sun et al., 2015). GTP-binding protein smg p21B (RAP1B), a RAS family member, can activate B-Raf and subsequently activate MEK via Raf-mediated phosphorylation. MEK can then phosphorylate and activate ERK. The MAPK/ERK pathway regulates the activities of several transcription factors including the v-ets avian erythroblastosis virus E26 oncogene homolog (ETS) (Yang et al., 1996; Seidel & Graves, 2002), which can bind to the MMP9 promoter and induce MMP9 transcription (Ghosh, Basu & Roy, 2012).

MMP9 has been shown to play an important role in many cancers. However, to our knowledge, the expression levels of MMP9 and the relative molecular signaling pathways of MMP9 have never been evaluated together in ccRCC. In the present study, we systematically investigated the expression level and prognostic value of MMP9 in ccRCC.

## Materials and Methods

### Extraction of clinical and microarray gene expression data from ccRCC patient datasets

In the research, ccRCC datasets were extracted from GEO database (http://www.ncbi.nlm.nih.gov/geo/) and TCGA database (http://www.cbioportal.org/data_sets.jsp). Seven datasets with more than 700 specimens, GSE40435 (Wozniak et al., 2013), GSE46699 (Eckelpassow et al., 2014), GSE15641 (Jones et al., 2005), GSE66272 (Wotschofsky et al., 2016), GSE36895 (Peñallopis et al., 2012), GSE53757 (Von Roemeling et al., 2014) and GSE14994 (Beroukhim et al., 2009) were obtained. First, the probe ID was converted into a gene symbol. When a gene was mapped to different probes, the genic expression value was calculated by the average expression value. Next, the data were translated into log2 logarithms, and the robust multichip averaging method was used to perform the median normalization (Irizarry et al., 2003). The clinical data and mRNA expression data of MMP9 in ccRCC were extracted from the TCGA database. According to the median value of gene or protein expression, samples were divided into low and high expression groups. We compared the clinical specimens of cancer vs. normal control datasets using the Student’s  *t*-test to generate a * P* value. A *P * value of <0.05 was considered statistically significant.

The prognostic values of the MMP9 mRNA expression for ccRCC patients were obtained from the TCGA database. The survival analyses were performed using the cutoff values of median MMP9 expression in ccRCC patients. According to the median value of gene expression, samples were divided into low and high expression groups.

### Patients and specimens

The research consisted of 202 samples from 101 patients with ccRCC. All the patients had a renal resection at the Fujian Provincial Hospital between January 2016 and June 2017. The standard requirements for patients included in the study were: (1) histologically confirmed ccRCC; (2) no history of other malignancy; (3) no prior neoadjuvant chemotherapy. The study was performed with the approval of the Ethics Committee of Fujian Provincial Hospital. Written informed consent was given by the patients for their information and specimens were stored in the hospital database and used for research.

### Immunohistochemical (IHC) staining

Paraffin blocks that contained sufficient formalin-fixed tumor specimens were serial sectioned at 3 µm and mounted on silane-coated slides for immunohistochemical staining analysis. The sections were deparaffinized with dimethylbenzene and rehydrated through 100, 100, 95, 85, and 75% ethanol. Antigen retrieval treatment was done in 0.01 mol/L sodium citrate buffer (autoclaved at 121 °C for 2 mins, pH 6.0) and endogenous peroxidase was blocked by incubation in 3% H_2_O_2_ for 10 mins at room temperature. The sections were washed in PBS solution subsequent and blocked with 10% goat serum (ZhongShan Biotechnology, China) for 30 mins and incubated with anti-MMP9 (ab38898, 1:100 dilution, abcam, polyclonal) at 4 °C for 12 h. The sections were washed in PBS solution three times and incubated with HRP-conjugated secondary antibody for 30 mins at room temperature. All slides were counterstained diaminobenzidine (DAB) solution and 20% hematoxylin, and dehydrated. The primary antibody diluent was regard as negative controls (Sun et al., 2017).

### Evaluation of immunostaining intensity

Immunohistochemical staining tissue sections were reviewed and scored by two independent pathologists. The score was calculated according to the proportion of stained tumor cells and intensity of cellular staining. The intensity of cellular staining was scored between 0 to 3. 0 equals no staining; 1 equals weak staining; 2 equals moderate staining and 3 equals strong staining. The proportion of stained tumor cells was scored between 1 to 4, 1 equals to 0–25%; 2 equals to 26–50%; 3 equals to 51–75% and 4 equals to 75–100%. The multiplication of these two variables was calculated as final score. The staining was divided into five grades according to the final score as follows: 0 score, 0; 1 score, 1–2; 2 score, 3–4; 3 score, 6–8; 4 score, 9–12.

### Statistical analysis

In the research, the student’s *t*-test was used to calculate the mRNA expression level in ccRCC tissues and adjacent normal renal tissues by using GraphPad-prism5. Log-rank test was used to calculate the survival analysis by using IBM SPSS version 19.0. Multivariate survival analysis was performed by using stepwise Cox proportional hazards regression model. All *P* value of <0.05 was considered statistically significant.

## Result

### The mRNA level of MMP9 is up-regulated in ccRCC

**Figure 1:**
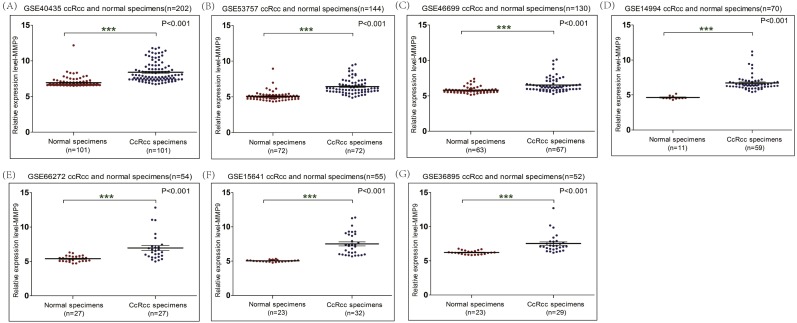
The mRNA level of MMP9 in ccRCC based on GEO database. The mRNA expression of MMP9 in ccRCC tissues and adjacent normal renal tissues were compared. Seven mRNA datasets were employed including GSE40435 (A), GSE53757 (B), GSE46699 (C), GSE14994 (D), GSE66272 (E), GSE15641 (F) and GSE36895 (G).

**Figure 2:**
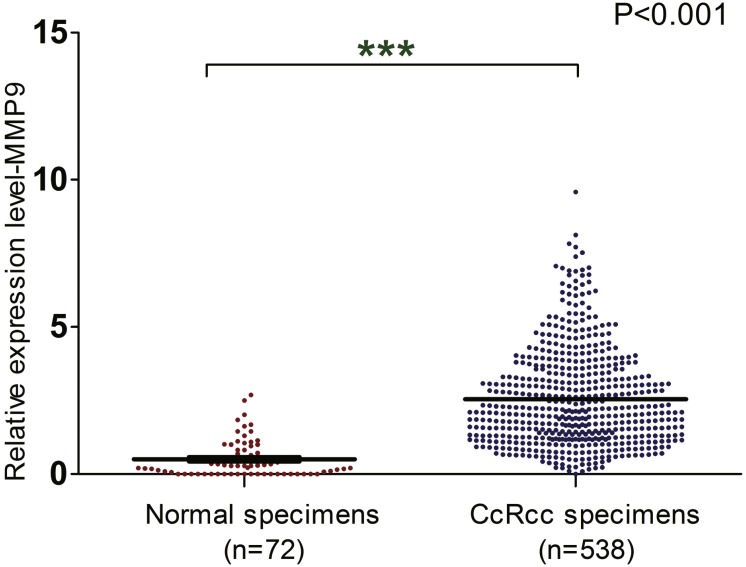
The mRNA level of MMP9 in ccRCC based on TCGA database. The mRNA expression of MMP9 in ccRCC tissues and adjacent normal renal tissues were compared using TCGA database.

In order to explore the expression of MMP9 in ccRCC patients, a total of seven related GEO datasets containing 353 patients were employed. Based on these datasets (GSE40435, GSE46699, GSE15641, GSE66272, GSE36895, GSE53757, GSE14994) (Supplemental Information 1), MMP9 was over-expressed in ccRCC tissues compared with adjacent normal renal tissues (Fig. 1). Meanwhile, the mRNA expression level of MMP9 was also up-regulated in ccRCC when the TCGA database was analyzed (Fig. 2) (Supplemental Information 2).

### The protein level of MMP9 is up-regulated in ccRCC

**Figure 3:**
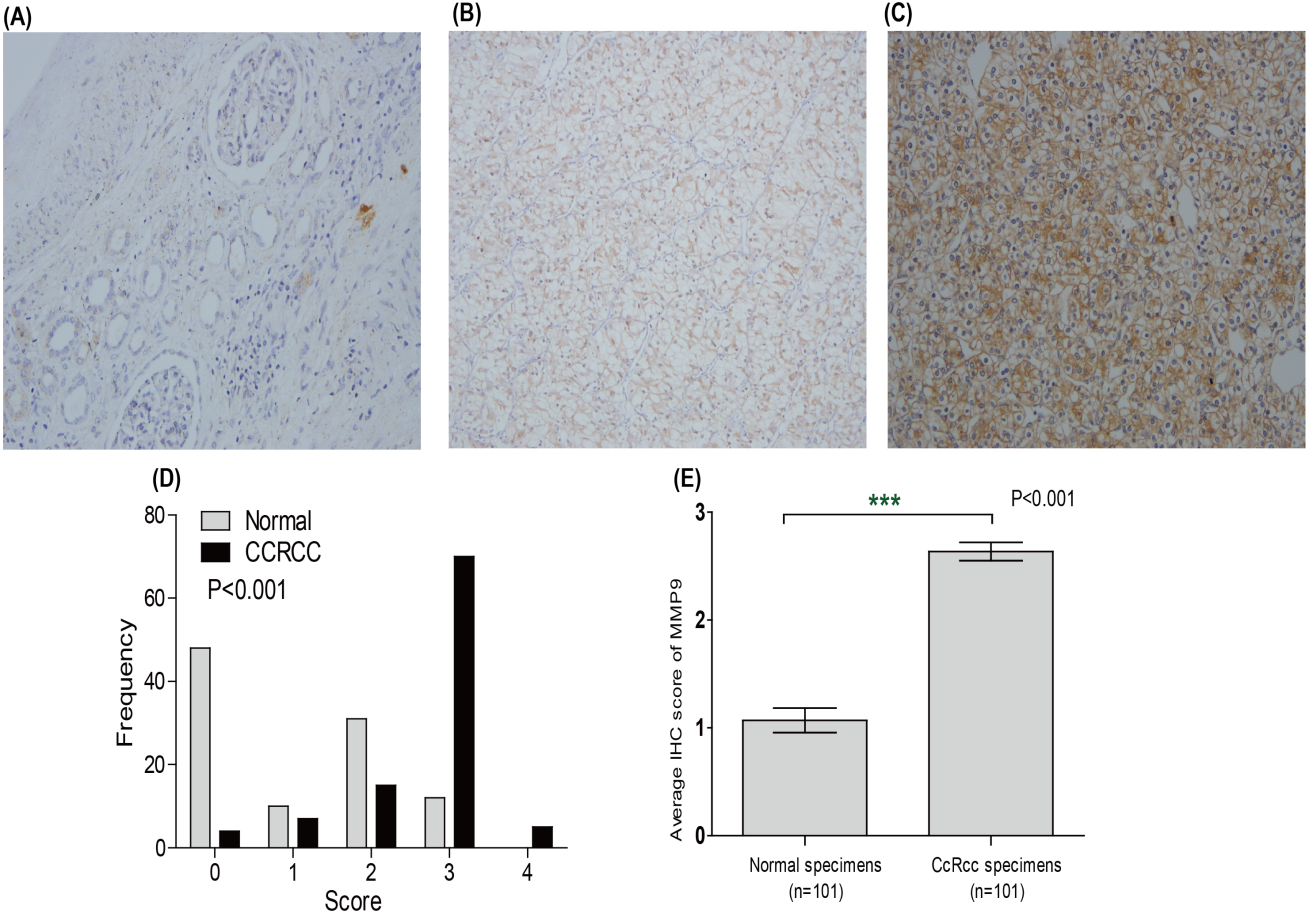
The protein level of MMP9 in ccRCC. The protein expression of MMP9 in ccRCC tissues and their adjacent normal renal tissues were compared using immunohistochemical staining. Representative adjacent normal renal tissues staining (A), ccRCC tissues staining (B), frequency distributions of proteins expression across the cohort (C), and the average score of immunohistochemical staining (D) were shown.

Moreover, IHC staining was used to analyze the protein levels of MMP9 in 101 ccRCC tissues and their adjacent normal renal tissues collected at Fujian Provincial Hospital. Representative staining and the frequency distributions of these scores were presented (Figs. 3A–3C). The mean scores of MMP9 proteins in ccRCC and adjacent normal renal tissues were 2.64 and 1.07, respectively (Fig. 3D).

**Figure 4:**
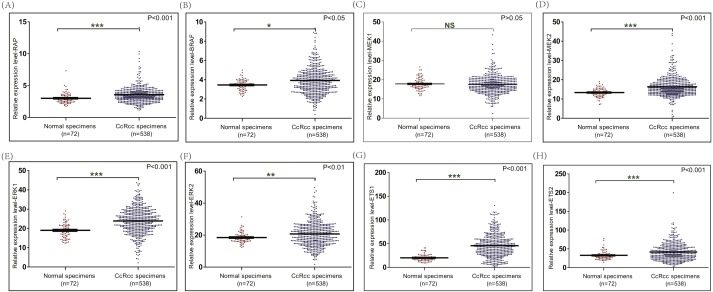
The mRNA level of RAP1B, BRAF, MEK1, MEK2, ERK1, ERK2, ETS1 and ETS2 in ccRCC based on TCGA database. The mRNA expression of RAP1B, BRAF, MEK1, MEK2, ERK1, ERK2, ETS1 and ETS2 in ccRCC tissues and adjacent normal renal tissues were compared using TCGA database.

### The mRNA levels of RAP1B, BRAF, MEK2, ERK1, ERK2, ETS1 and ETS2 were up-regulated in ccRCC

The RAP1B/BRAF/MEK/ERK/EST signaling pathway can regulate the expression level of MMP9. The increased mRNA expression level of RAP1B, BRAF, MEK2, ERK1, ERK2, ETS1 and ETS2 was observed in ccRCC using TCGA database (Fig. 4)

### Overexpression of MMP9 is an unfavorable prognostic factor

In the TCGA ccRCC cohort, we observed that patients with advanced stage and high expression level of MMP9 were at significantly increased risk of death. Patients with age >60, laterality = left also have a high risk of death (Table 1).

**Table 1:** Univariate analysis of the correlation between clinicopathological parameters and survival of ccRCC patients in TCGA cohor.

Variables	Patients (*n*)	MST (days)	Log-rank test	*P*
**Age (years)**				
	≤60	263	NA		
	>60	263	1,964	11.83	0.001^a^
**Gender**				
	female	184	2,343		
	male	342	2,299	0.098	0.755
**Laterality**				
	left	248	2,227		
	right	277	NA	5.517	0.019^a^
**Race**				
	white	456	2,343		
	other	63	1,913	0.424	0.515
**Tumor stage**				
	I/II	320	2,764		
	III/IV	203	1,200	85.124	0.000^a^
**MMP9 expression**				
	Low	263	2,764		
	High	263	1,912	14.992	0.001^a^

**Notes.**

a *P* < 0.05, statistical significance, MST, median survival time, NA, not available.

**Figure 5:**
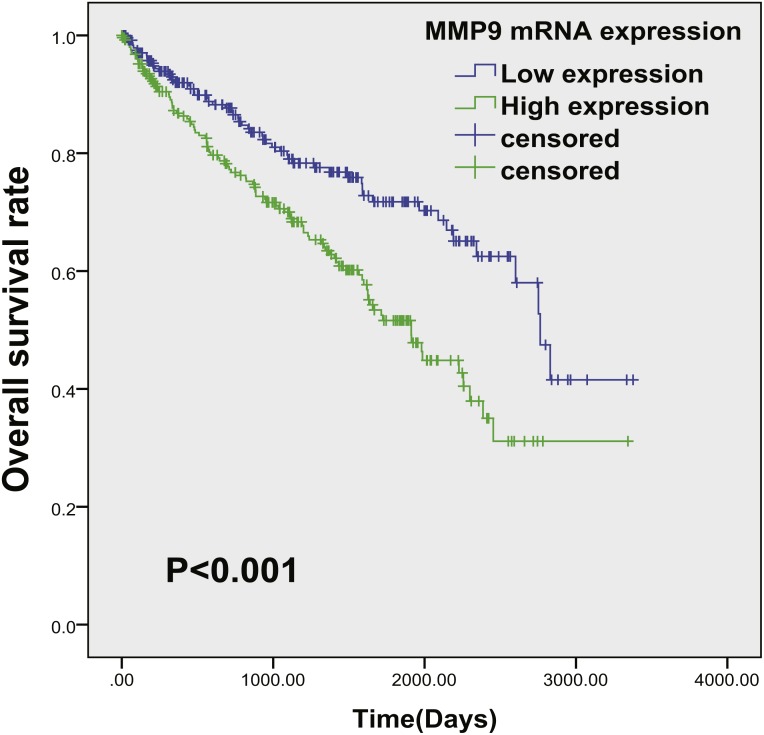
The prognostic value of MMP9 in ccRCC. The Kaplan–Meier survival analyses of MMP9 mRNA expression of the overall survival time of ccRCC patients using the TCGA database.

**Table 2:** Multivariate analysis of the correlation between clinicopathological parameters and survival of ccRCC patients in the TCGA cohort.

Covariates	Standard error	HR	95% CI for HR	*P*
Age (≤60 vs >60)	0.167	0.615	0.443–0.853	0.004^a^
Laterality (left vs right)	0.162	1.510	1.099–2.074	0.011^a^
Tumor Stage (I/II vs III/IV)	0.177	0.239	0.169–0.338	0.000^a^
MMP9 (low vs high)	0.172	0.660	0.471–0.924	0.015^a^

**Notes.**

a *P* < 0.05, statistical significance.

The survival analysis of MMP9 mRNA was shown in Fig. 5. We found that the high mRNA expression of MMP9 correlated with an unfavorable clinical outcome of ccRCC patients (Supplemental Information 3), after adjusting for tumor location, stage and patient age, gender and race (Table 2).

## Discussion

In this study, the expression and prognostic relevance of MMP9 in ccRCC were examined. Our analysis involved eight datasets with more than 1,300 samples from GEO and TCGA databases and the IHC analyses on one local ccRCC patient cohort (*n* = 101). A higher MMP9 mRNA level was observed in ccRCC tissues compared with adjacent normal renal tissues using the GEO database (Fig. 1) and the TCGA database (Fig. 2). At the same time, a higher protein expression level of MMP9 was observed in the 101 ccRCC tissues compared with their adjacent normal renal tissues using IHC. The mean scores of MMP9 proteins in ccRCC and adjacent normal renal tissues were 2.64 and 1.07, respectively (paired *t* test: *t* = 9.891, *df* = 100, *p* < 0.001; *f* test: *f* = 12.478, *p* < 0.001). The increased MMP9 expression was significantly associated with poor prognosis of ccRCC patients (HR = 0.66, 95% CI [0.471–0.924], *p* = 0.015).

**Figure 6:**
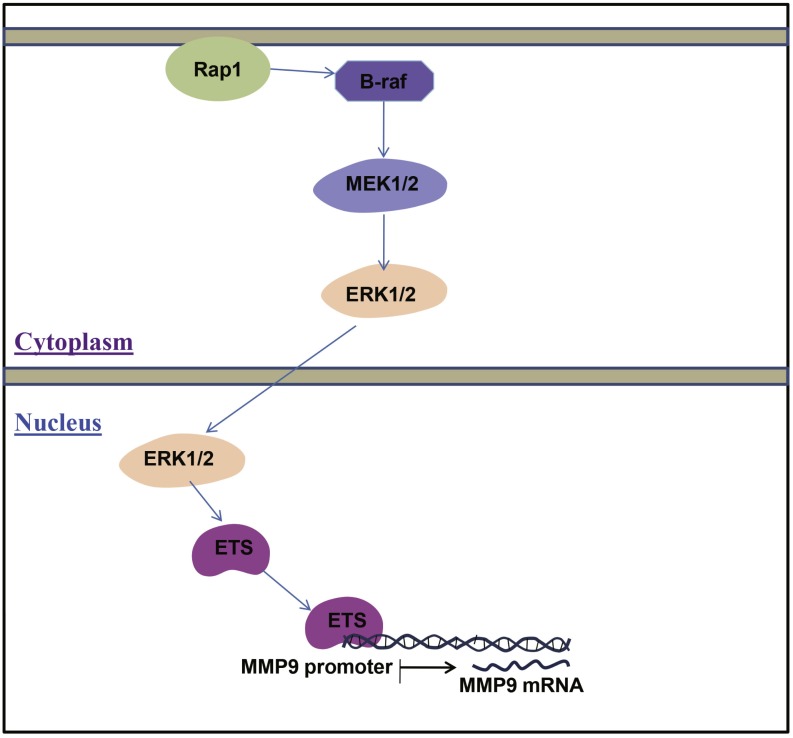
A diagram illustrating the MAPK/ERK/ETS signaling pathway regulated MMP9 in ccRCC. In ccRCC, Rap1 activates the B-Raf, Raf kinase phosphorylates and activates MEK. MEK phosphorylates and activates ERK. The MAPK/ERK pathway regulates the activities of several transcription factors including ETS. ETS transcription factor can bound to MMP9promoter and active MMP9.

According to our results, the mRNA and protein expression levels of MMP9 were enhanced in ccRCC tissues compared with adjacent normal renal tissues. As we know, the expression level of MMP9 can be regulated by MAPK/ERK/ETS signaling pathway. In order to explore the potential mechanism responsible for the increased expression level of MMP9 in ccRCC, the upstream factors of MMP9 such as RAP1B, BRAF, MEK2, ERK1, ERK2, ETS1 and ETS2 were detected. RAP1B (*P* < 0.001), BRAF (*P* < 0.05), MEK2 (*P* < 0.001), ERK1 (*P* < 0.001), ERK2 (*P* < 0.01), ETS1 (*P* < 0.001) and ETS2 (*P* < 0.001) were enhanced in ccRCC compared with normal renal tissues (Fig. 4). These results suggested that the increased level of MMP9 may be caused by the activation of the MAPK/ERK/ETS signaling pathway in ccRCC (Fig. 6). However, it is just a potential mechanism. More studies are clearly required to further prove this hypothesis.

MMP9 is able to degrade type IV collagen, thereby facilitating stromal and vascular invasion by tumor cells. Our study implied that MMP9 inhibitors may have clinical efficacy for ccRCC patients, as MMP9 was overexpressed in ccRCC and associated with poor prognosis. Further studies would be needed to verify this supposition and MMP9 may be a new promising therapeutic target for ccRCC.

## Conclusion

Collectively, our data showed that the mRNA and protein levels of MMP9 were enhanced in ccRCC, and the increased level of MMP9 may be due to the activation of the MAPK/ERK signaling pathway. Moreover, high expression of MMP9 in ccRCC was associated with the poor prognosis (Fig. 5). These results suggested that MMP9 might be an important oncogene in ccRCC and presented a potential therapeutic target.

##  Supplemental Information

10.7717/peerj.5050/supp-1 Supplemental Information 1 The expression value of MMP9 extracted from GEO database

10.7717/peerj.5050/supp-2 Supplemental Information 2 The expression value of MMP9 extracted from TCGA database

10.7717/peerj.5050/supp-3 Supplemental Information 3 The data of survival analysis extracted from TCGA database

## Additional Information and Declarations

### Data Availability

The following information was supplied regarding data availability: The raw data are provided in the Supplemental Files.
